# Surdity in the OR: An Unusual Case of Brainstem Anesthesia

**DOI:** 10.1155/2017/4645381

**Published:** 2017-01-09

**Authors:** Howard D. Palte, Don P. Hoa, Aldo Pavon Canseco

**Affiliations:** Department of Anesthesiology, Miller School of Medicine, University of Miami, Miami, FL, USA

## Abstract

Brainstem anesthesia is a potentially life-threatening complication of regional ophthalmic anesthesia. This case report chronicles an unusual presentation of brainstem anesthesia following an eye block. The unique features of this case were the presenting symptoms of deafness and slurred speech in the absence of loss of consciousness, respiratory depression, or contralateral ophthalmoplegia. This report underscores two key points: first, the importance of ongoing patient monitoring after performance of an eye block; second, the exigency of supportive therapy in suspected cases of brainstem anesthesia.

## 1. Introduction

Brainstem anesthesia is a potentially life-threatening complication of regional ophthalmic anesthesia. The classic presentation is one of altered states of consciousness in association with permutations of impaired respiratory drive, hemodynamic instability, and contralateral ophthalmoplegia. In general, symptoms manifest only 5 to 20 minutes after completion of the block. We present an atypical case of brainstem anesthesia presenting with hemodynamic volatility, deafness, and slurred speech. In addition, this case was unusual because consciousness was preserved and respiratory effort was not impaired.

## 2. Case Report

A 42-year-old male sustained a prior penetrating injury to his left eye and now presented for a corneal amniotic membrane graft. He had a background history of hypertension and diabetes mellitus. Current medications included metoprolol, lisinopril, and metformin. Fasting blood sugar on admission was 223 mg%. The patient was transported to the holding area on a chair-bed and standard ASA monitors, peripheral IV, and nasal oxygen cannula were placed. Vital signs included BP122/83 mmHg, pulse 90/min, and S_p_O_2_ 99%. Following sedation with midazolam (2 mg) and fentanyl (100 mcg), a left-sided inferotemporal transconjunctival eye block was performed using a 27 G 31 mm needle, maintaining the eye in neutral gaze. Nine mL of a local anesthetic (LA) mixture containing lidocaine 2%, ropivacaine 1%, and Hylenex® 7.5 IU/mL was administered. The performance of the block was uneventful. Ten minutes later the patient developed paroxysmal tachycardia (140/min) and acute, severe hypertension (240/140 mmHg) that was immediately treated with intravenous nicardipine (200 mcg) and labetalol (10 mg). At this point, the patient was somewhat somnolent but responded appropriately to verbal command. However, he displayed signs of mild left-sided facial weakness. Within five minutes of treatment, the blood pressure and heart rate returned to baseline levels. The peripheral oxygen saturation (S_p_O_2_) was always ≥98%. His condition was assessed as stable and he was transferred to the OR.

In the OR monitors were reapplied and the patient was asked to shift towards the head of the bed. He was now unresponsive to verbal command but opened his eyes on manual stimulation and indicated that he was unable to hear. His vital signs remained stable with no evidence of respiratory embarrassment or decline in S_p_O_2_. In addition to deafness, a repeat neurological examination revealed left-sided facial weakness, slurred speech, and an inability to protrude the tongue. Although the contralateral pupil was dilated, the light reflex and extraocular muscle function remained intact. The blood glucose was reassessed at 271 mg%.

At this stage, the differential diagnosis included brainstem anesthesia and cerebrovascular accident secondary to hypertensive crisis. The planned surgery was aborted and the patient was transferred to a tertiary center for an urgent CT scan to exclude stroke. This was performed within the recommended time constraint for stroke victims and revealed no intracranial pathology. Within three hours there was full return of neurologic integrity, including hearing. A full disclosure of the events was made to the patient. He was discharged home later the same day.

## 3. Discussion

Brainstem anesthesia is a rare, potentially life-threatening complication of regional ophthalmic anesthesia. The true incidence varies according to the source quoted. However, its prevalence may be on the decline because traditional retrobulbar anesthesia has now been largely superseded by less invasive peribulbar techniques. For example, Riad and Akbar reported only one episode in a case series of over 33000 eye blocks (0.002%) [1], whereas Kumar and Dowd pegged the incidence at 0.03% [2]. However, they opined that the incidence could be as high as 0.8% when retrobulbar blocks are performed with long needles. Lee et al. evaluated the American Society of Anesthesiology (ASA) closed claims database for complications associated with eye blocks from 1980 through 2000 [3]. They found only one claim with cardiorespiratory arrest attributable to eye block although there were another seven instances where cardiac or respiratory arrest followed an eye block. However, the concurrent use of sedation confounded the etiology.

In North America the majority of adult ophthalmic surgical interventions are conducted under topical or regional anesthesia, mostly needle-based techniques. Conversely, in the United Kingdom and Europe, sub-Tenon's block is favored due to the contention that it eliminates risk of needle misadventure. However, no regional technique is entirely safe and major complications have been reported with all techniques [4–7].

Brainstem anesthesia develops secondary to injection of LA within the dural sheath that surrounds the intraorbital segment of the optic nerve. Local anesthetic is subsequently transported towards the optic chiasm and brainstem. Traditionally, the presenting signs include a combination of altered mentation (agitation, confusion, and unresponsiveness), apnea, cardiovascular collapse (hypotension, bradycardia, or asystole), and shivering in association with ophthalmoplegia and amaurosis in the contralateral eye. Convulsions may occur secondary to hypoxia. Typically, symptoms begin 5–10 minutes after the block but presentation may be delayed up to 20 minutes. In general, most cases have favorable outcomes with no long-term sequelae provided that assisted-ventilation and indicated inotropic support are administered.

There is ample evidence that LA injected within the optic nerve dural cuff reaches the brainstem. In 1969, Reed et al. performed orbitography on a patient with an intraorbital tumor and demonstrated injected radiopaque dye within the intracranial subdural space [8]. Wang et al. injected cadaver optic sheaths with methylene blue and traced progression of the dye as far as the middle cranial fossa [9]. In a cadaver model, Drysdale placed a needle within the optic sheath and injected 3 mL radiopaque dye [10]. He demonstrated that contrast material tracked proximally along the nerve to the optic chiasm, ultimately reaching the pons and midbrain. Finally, Kobet retrieved high levels of lidocaine and bupivacaine in the CSF of a case of brainstem anesthesia following retrobulbar block [11].

**Figure 1:**
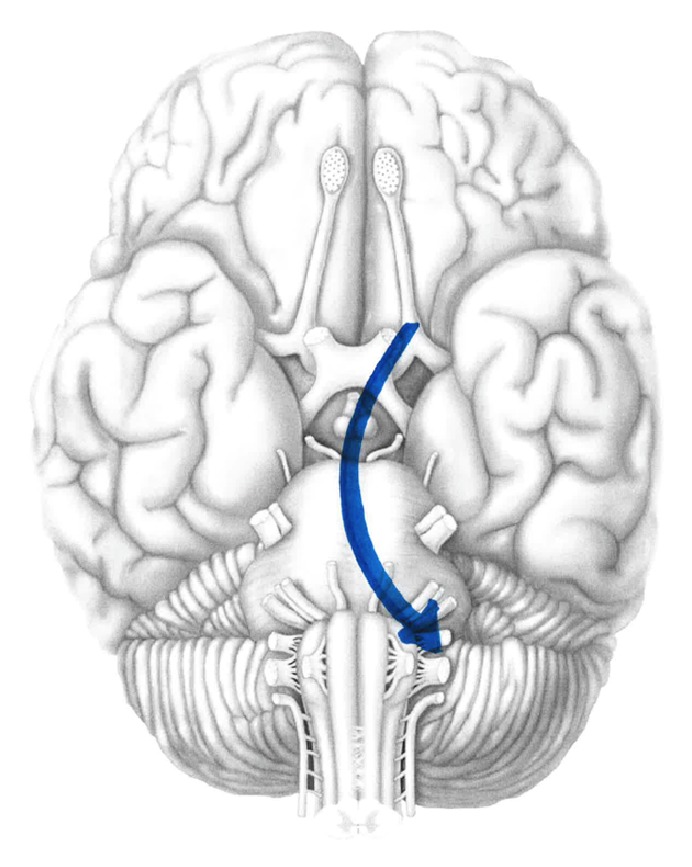
Diagrammatic representation of local anesthetic spread along brainstem from CN II to CN VIII.

The clinical course of this case was atypical. The common features of loss of consciousness, respiratory depression, hypotension and contralateral ophthalmoplegia, and amaurosis were absent. Instead, the dominant signs were transient malignant hypertension, hearing loss, and slurred speech. Moreover, pupillary dilation was the only sign in the contralateral eye. We hypothesize that the initial insult was needle penetration of the lateral segment of the dural sheath. Thereafter, local anesthetic tracked in a subdural plane over the lateral optic chiasm and hypothalamus producing a block in parasympathetic efferent activity and overexpression of symapthetic activity and manifesting as hypertensive crisis. We postulate that LA remained confined within this compartment of the optic nerve because there was neither ophthalmoplegia nor amaurosis in the contralateral eye. Ultimately, LA blocked cranial nerves VIII and XII producing deafness and slurred speech (Figure 1). Furthermore, this case is peculiar because brainstem anesthesia followed use of a 31 mm needle. Katsev et al. measured orbital depth in a series of skulls and recommended that 31 mm needles afford greater protection against optic sheath penetration than commonly used 38 mm needle [12].

The peribulbar block deposits LA outside the extraocular muscle cone away from the globe and vital structures. Since successful anesthesia depends on passive diffusion of local anesthetic into the retrobulbar compartment, the technique requires administration of a greater LA volume than for the traditional retrobulbar block. One disadvantage of this technique is that the ultimate needle-tip position in relation to the muscle cone is unconfirmed, raising the specter of intraconal injection. In this case, we contemplate that posttraumatic scar tissue within the orbital cavity produced an aberration of the muscle cone with lateral displacement of the optic nerve.

This case underscores several important take-home points. First, brainstem anesthesia remains a real hazard of regional ophthalmic blockade and may present with atypical features. Second, the latent period between block and symptomatology is variable and influenced by many factors including orbital anatomy, volume of local anesthetic, rate of spread, and constitution of the LA. Also, a prior history of eye trauma or repeat surgeries raises the specter of possible intraorbital scarring and anatomic aberration. Third, the differential diagnosis should include other acute neurologic or cardiovascular insults that produce similar symptomatology, especially cerebrovascular accident. Fourth, beneficial outcomes with no long-term sequelae are expected when assisted-ventilation and inotropic support are instituted in a timely manner. Finally, the temporal relationship between performance of an eye block and onset of neurological, cardiovascular, or respiratory symptomatology should raise suspicion of brainstem anesthesia.
